# Emissivity Correction and Thermal Pattern Reconstruction in Eddy Current Pulsed Thermography

**DOI:** 10.3390/s23052646

**Published:** 2023-02-28

**Authors:** Kongjing Li, Gui Yun Tian, Junaid Ahmed

**Affiliations:** 1 Research and Development Centre, Dynex Semiconductor, Lincoln LN6 3LF, UK; 2 School of Automation Engineering, University of Electronic Science and Technology of China, Chengdu 610054, China; 3 School of Engineering, Newcastle University, Newcastle upon Tyne NE1 7RU, UK; 4 Computer Systems Engineering Department, Sukkur IBA University, PRG9+PM, Sukkur 65200, Pakistan

**Keywords:** thermography, emissivity correction, thermal pattern reconstruction, intelligent fault diagnosis

## Abstract

Emissivity variations are one of the most critical challenges in thermography technologies; this is due to the temperature calculation strongly depending on emissivity settings for infrared signal extraction and evaluation. This paper describes an emissivity correction and thermal pattern reconstruction technique based on physical process modelling and thermal feature extraction, for eddy current pulsed thermography. An emissivity correction algorithm is proposed to address the pattern observation issues of thermography in both spatial and time domains. The main novelty of this method is that the thermal pattern can be corrected based on the averaged normalization of thermal features. In practice, the proposed method brings benefits in enhancing the detectability of the faults and characterization of the materials without the interference of the emissivity variation problem at the object’s surfaces. The proposed technique is verified in several experimental studies, such as the case-depth evaluation of heat-treatment steels, failures, and fatigues of gears made of the heat-treated steels that are used for rolling stock applications. The proposed technique can improve the detectability of the thermography-based inspection methods and would improve the inspection efficiency for high-speed NDT&E applications, such as rolling stock applications.

## 1. Introduction

In recent years, thermography-based non-destructive testing and evaluation (NDT&E) techniques have been developed rapidly in industrial applications. With the benefits of contactless and large inspection areas, high testing efficiency can be achieved by using active thermography-based NDT&E methods. However, the emissivity issue is always the most critical challenge of thermography-based technologies. These range from difficulties regarding the evaluation of thermal patterns illustrated; materials with high reflectivity surfaces, materials with low emissivity surfaces, and objects where surfaces are formed from different materials (and as such, different emissivity values). Thermography also provides relative temperature maps, which can provide false temperature contrasts as a result of different emissivity across the sample under test (SUT). Non-uniform emissivity across sample surfaces poses challenges in showing accurate thermal contrast from the infrared detector. The material information in a thermal image is obscured by false temperature/thermal variations due to non-uniform emissivity.

As an emerging NDT&E technology, eddy current pulsed thermography (ECPT) measurements contain information from multiple physical factors, which have been widely used in the material evaluation and characterization for a wide range of conductive materials [1,2,3]. The infrared image sequences contain valuable information in both the spatial and time domains. With this technique, the infrared radiation emitted by the SUT surface, generally referred to as the spectral radiant emittance (in W/m^2^ μm), at a given temperature (Planck’s law) is captured, producing the surface thermal pattern of the SUT. Since the ECPT material evaluation is based upon evaluations of the temperature contrast within infrared image sequences, if the output signals of an infrared camera differ with varied surface conditions across the same material, even with the same actual temperature in these areas, the evaluation of material is affected. In general, the emissivity correction can involve external apparatus, such as specific optical lenses, special preparation of the SUT, optimized radiation reduction chambers, and the use of surrounding references with known temperatures or emissivity values (crinkled aluminum foil as 0 and known values of high emissivity paints which are normally close to 1) [4,5,6,7]. To obtain accurate thermal contrast in ECPT more conveniently, and to reduce the influences of emissivity variation efficiently and practicably in real-world scenarios, it is necessary to implement pre-processing of the infrared image sequences directly from the output signal of the infrared camera. As it is sufficient to have information regarding thermal variation in ECPT for material evaluation, we do not require the true values of the emissivity across the surface of the SUT. This drives us to find a more reliable, convenient, and general method for emissivity correction.

Here, we introduce a novel emissivity correction method for minimizing the effect of an emissivity variation. Unlike prior research on emissivity correction which is based on surface coating or phase information extraction from pulsed phase thermography (PPT) [8,9,10], principal component thermography (PCT) [11,12], or other normalization methods [13,14], the proposed method keeps the original thermal contract information and presents the same temperature-based thermal patterns as the SUT. Experimental studies have been carried out on two groups of samples: case-carburized gear steels with an oil-coated surface, and case-nitride gear steels with varied case depths and emissivity values. The results validated our emissivity correction method, which showed that the informative value of ECPT has been improved significantly. It has also been demonstrated that ECPT can be employed to separate the depth information of case-hardened gear steels. Further validations of the proposed emissivity correction method are conducted on gear samples that have been through long-term fatigue tests. Both fatigue failure and fatigue progress are successfully revealed after emissivity correction in thermographic images. The rest of the paper is organized as follows: The physical process modelling and the emissivity correction in eddy current pulsed thermography are introduced in Section 2. Experimental study and validations are illustrated in Section 3. Finally, the conclusion and uncertainty analysis are drawn in Section 4.

## 2. Physical Process Modelling of Eddy Current Pulsed Thermography

To implement thermal pattern reconstruction, a series of information processing algorithms are delivered. Firstly, the theoretical background of the electromagnetic thermography sensing system is introduced. Secondly, based on an understanding of the mathematical and physical modelling of the electromagnetic thermography system, the emissivity correction algorithm is proposed.

### 2.1. Eddy Current Pulsed Thermography

**Figure 1:**
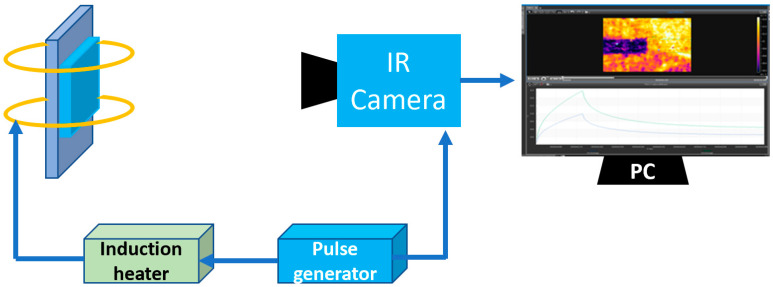
ECPT system.

Eddy current pulsed thermography (ECPT) employs a high-frequency alternative magnetic field as excitation induced by a coil-carried alternating current, as shown in Figure 1. A conductive material is then inductively heated by the induced eddy currents on it. An infrared (IR) camera utilizing an active cooled 1.5–5.1 μm InSb detector and induction heater are simultaneously triggered to record thermal videos which include a pulse induction heating stage and a cooling stage. For the ECPT experimental studies implemented in this paper, the frequency is set to 268 kHz, and the samples are heat-treated steels with different carbonizing and nitriding times. Considering the skin effect of eddy currents on inductive materials, more than 95% of the induced eddy currents exist in materials within the penetration depth; this is about 1 μm in most steels, thus, it can be considered that the Joule heat generated by the eddy current is concentrated on the surface of materials. Since it considers only the small heating region under and along the excitation coil in ECPT material evaluations, it can be assumed that Joule heating is uniformly generated in this area (about 1.5 mm width in this paper, the value depending on coil diameter and lift-off distance), all material parameters are constant during one inspection period (about 1 s including 200 ms heating time and 800 ms cooling time), and the mathematical model of heat transfer can be built by the micro-element method in the inspection area. The thermal equilibrium of an infinitesimal body in eddy current penetration depth can be discussed here. 

### 2.2. Emissivity Correction Algorithm

The IR detector produces signals which are proportional to the photon flux emitted from position (*x*, *y*) on the SUT surface. This is normally referred to as spectral radiance (*R*). According to Planck’s law, the relationship between the temperature *T*(*x*, *y*, *i*) and its black body spectral radiance *R_BB_* (*x*, *y*, *i*, *λ*, *T*) which in the unit [W/m^3^] is:

(1) $$R_{BB}\left(x,y,i,\lambda ,T\right)=\frac{2\pi hc^{2}}{\lambda^{2}\left[exp\left(\frac{hc}{\lambda cT\left(x,y,i\right)}\right)-1\right]}$$

where *i* is one frame of the infrared video, *c* is the speed of light in vacuum, *h* is Planck’s constant, *c* is Boltzmann’s constant, *T* is the temperature in Kelvin, and the *λ* is the wavelength in meters. Since the temperature variations are small in comparison to the pulsed excitation, we assume that emissivity is independent of temperature, and the wavelength does not change significantly during the measurement. We also have to take into account the emissivity *ε*(*x*, *y*) when the SUT is considered a grey body.

(2) $$R_{Grey}(x,y,i,\lambda ,T)=\varepsilon \left(x,y\right)R_{BB}(x,y,i,\lambda ,T)$$

The measured radiance *R_M_* (*x*, *y*, *i*, *λ*, *T*) received by the detector is the combination of four radiance sources: (1) lenses and filter, (2) the capture environment around the SUT, (3) infrared detector itself (Narcissus effect) and (4) the emitted spectral infrared radiance from the SUT during heating.

The thermal detector cell consists of an absorber connected to a heat sink (enabling a constant temperature at the cell); as this absorber absorbs radiation, its temperature rises above that of the heat sink. A thermometer can be attached to the absorber, allowing the absorbed power to be calculated from the measured temperature. Finally, the readout circuit converts the analog output signal of the detector to a 12-bit parallel digital level signal at the output of the processing unit of the IR camera. The output thermal patterns of the camera are a function of *R_M_* (*x*, *y*, *i*, *λ*, *T*) and the general performance of the thermal detector, and are given by

(3) $$TP\left(x,y,i\right)=\varepsilon \left(x,y\right)R_{M}\left(x,y,i,\lambda ,T\right)$$

Such that the output signal *TP*(*x*, *y*, *i*) from a non-ideal body is related to the signal *TP_BB_*(*x*, *y*, *i*) from a blackbody by

(4) $$\varepsilon \left(x,y\right)\cdot TP_{BB}(x,y,i)+TP_{BG}(x,y,i)=TP(x,y,i)$$

where *ε*(*x*, *y*) ⋯ *TP_BB_*(*x*, *y*, *i*) is the fourth source of radiance received by the detector, the spectral infrared radiance from the SUT. *TP_BG_* (*x*, *y*, *i*) consists of the combined signal from the remaining three sources as explained above.

As one of the advantages of electromagnetic thermography, infrared video recording and eddy current excitation are synchronized. The first frame of the video is under the influence of all four sources of radiance received by the detector. Hence, the *TP_BG_*(*x*, *y*, *i*) can be removed by the subtraction of the first frame from the remainder, producing *TP_D_*(*x*, *y*, *j*),

(5) $$TP_{D}(x,y,j)=TP(x,y,i)-TP(x,y,0)=\varepsilon (x,y)TP_{DBB}(x,y,j)$$

where *TP_DBB_*(*x*, *y*, *j*) is proportional to *TP_BB_*(*x*, *y*, *j*). A true emissivity map *ε*(*x*, *y*) can be illustrated as:

(6) $$\varepsilon (x,y)=\frac{TP_{D}(x,y,j)}{TP_{DBB}(x,y,j)}$$

However, it is not available to acquire the precise temperature of a blackbody in real-world applications. For electromagnetic thermography emissivity correction, it is sufficient to have the relative emissivity across the SUT surface:

(7) $$\frac{\varepsilon \left(x,y\right)}{E\left[\varepsilon \left(x,y\right)\right]}=\frac{\frac{TP_{D}\left(x,y,j\right)}{TP_{DBB}\left(x,y,j\right)}}{E\left[\frac{TP_{D}\left(x,y,j\right)}{TP_{DBB}\left(x,y,j\right)}\right]}$$

where the denominator denotes an average value over all frames of each pixel, which is based on the time average theorem. *TP_DBB_*(*x*, *y*, *j*) is independent of (*x*, *y*) by definition. *TP_DBB_*(*x*, *y*, *j*) in the denominator can be taken out of the average. This results in

(8) $$\frac{\varepsilon \left(x,y\right)}{E\left[\varepsilon \left(x,y\right)\right]}=\frac{TP_{D}\left(x,y,j\right)}{E\left[TP_{D}\left(x,y,j\right)\right]}$$

which is independent of blackbody reference information. It can also be expressed as:

(9) $$\left\{TP_{D}\left(x,y,j\right)\right\}=\frac{E\left[\varepsilon \left(x,y\right)\right]}{\varepsilon \left(x,y\right)}=TP_{D}\left(x,y,j\right)$$

Hence, we can adapt the average value over all frames of every pixel *TP_A_*(*x*, *y*) as the relative emissivity map of *TP_D_*(*x*, *y*, *j*)

(10) $$TP_{A}\left(x,y\right)=E\left[TP_{D}\left(x,y,j\right)\right]$$

where *TP_A_*(*x*, *y*) indicates the coefficient which is proportional to the surface emissivity. The transient response is divided by *TP_A_*(*x*, *y*), namely

(11) $$TP_{N}(x,y,j)=\frac{TP_{D}(x,y,j)-TP_{D}(x,y,0)}{TP_{A}(x,y)-TP_{D}(x,y,0)}$$

**Figure 2:**
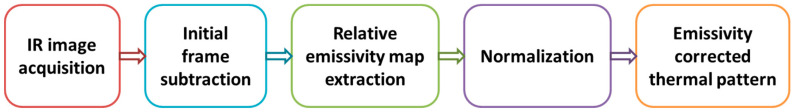
Flow chart of thermal pattern emissivity correction.

Equation (11) indicates that the proposed adjustment amounts to normalization by simply dividing the thermal response rise at each pixel by its average temperature rise. Hence, we can have the corrected surface thermal pattern after the emissivity map adjustment. The implementation of emissivity correction can be illustrated in the flow chart in Figure 2. To validate the proposed method of emissivity correction, we have applied it to two groups of samples. The first one is a sample of case-carburized gear steel coated with oil, where the oil introduces the issue of emissivity non-uniformity. The second group comprised four samples of case-nitrided gear steels with different emissivity values to each other, which were treated at different times, resulting in different case depths.

## 3. Results of the Experimental Study of Material Analysis and Gear Fault Detection

Experimental validations for the proposed methods are conducted in three aspects: (1) reducing the surface-oil-coating influence in carburizing-treated steel samples; (2) case-depth separation in nitriding-treated gear steels; (3) fault detection in gears and (4) fatigue process analysis of gears in realistic working scenarios.

### 3.1. Emissivity Correction and Case-Depth Separation

**Figure 3:**
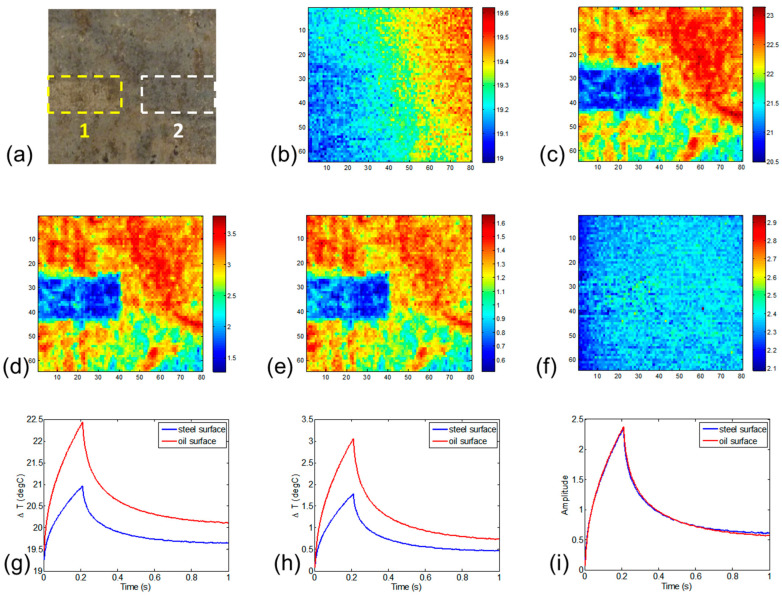
(**a**) Sample photograph, area 1 indicates the true surface of the steel, area 2 is the portion of the oil covered area equal in size to area 1. (**b**) Infrared image at room temperature. (**c**) Original ECPT image at maximum heating time. (**d**) Subtracted first frame. (**e**) Relative emissivity map. (**f**) Thermal pattern after emissivity correction. (**g**) Original thermal responses. (**h**) Post first frame subtraction. (**i**) Emissivity-adjusted thermal responses.

Figure 3 shows the emissivity correction procedure of the proposed method.

Figure 3a illustrates the variations in emissivity on the surface of the case-carburized gear steel sample, which is coated with oil. One small rectangular region, marked as area 1, has had oil removed to expose the true surface of the steel. Figure 3b shows the infrared image of the sample at room temperature. The thermal pattern is distorted by the environmental infrared effects of the background at the same temperature across the sample surface. Figure 3c shows the original ECPT image at the maximum heating time. The thermal pattern is extremely affected by the variation in surface emissivity caused by the coating oil, where the temperature of each pixel in the image should be approximately the same. To remove the environmental effects, the first frame has been subtracted from every subsequent frame, producing Figure 3d. To adjust the non-uniform emissivity, the relative emissivity map, illustrated by Figure 3e, is extracted by averaging the value of each pixel over all frames. Figure 3f shows the reconstructed thermal pattern after the emissivity correction, which is produced by normalization with the aid of the relative emissivity map. To compare the differences between the original infrared images and emissivity-corrected images, Figure 3g–i illustrates the transient thermal responses of the average value of areas 1 and 2, as shown in Figure 3a. Figure 3g illustrates the comparison between two areas with different emissivity values, which resulted in different thermal patterns under the same absolute temperature. Figure 3h shows the relative thermal responses after subtracting the first frame. Figure 3i shows the reconstructed transient thermal responses created with the aid of emissivity correction. Since the thermal pattern has been normalized, the vertical axis is the amplitude of normalized factors rather than temperatures. It shows that after emissivity correction, the transient thermal responses of two areas with the same value and different emissivity values match with each other.

**Figure 4:**
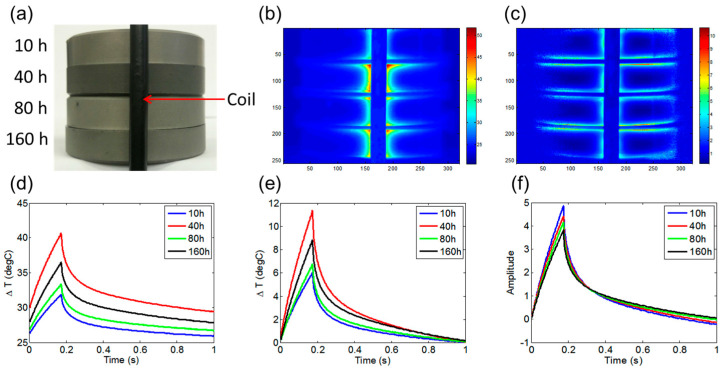
Images of four case-nitrided gear steels: (**a**) Sample photograph. (**b**) Original IR image at the maximum heating time. (**c**) Thermal pattern after emissivity correction. (**d**) Original transient temperature responses. (**e**) Post first frame subtraction. (**f**) Emissivity-adjusted transient thermal responses.

We also employed ECPT to separate different case depths of case-hardened steels, as depth information could be retrieved from thermal pattern variations. As illustrated in Figure 4a, the four case-nitride gear steels have been treated for different periods of 10, 40, 80, and 160 h. Each of them presents a different emissivity, most notably the 40-h sample, which presents a higher emissivity than the others. These differences in emissivity will introduce inaccurate temperature responses of the samples under ECPT. Figure 4b shows that the original infrared images at the temperature responses of the samples are different from each other and are not monotonically related to their treatment time. Figure 4c shows the reconstructed thermal pattern of four samples after emissivity correction. The higher edge temperature is due to the eddy current edge effect. The average values of small areas (5 × 3 pixel matrix) at the same positions of each sample close to the coil have been sampled. Figure 4d shows the original transient temperature responses. It shows that the measured start temperatures of each sample are different, as expected. This is due to the variation in emissivity and the infrared variations in the background environment across measurement times. To cancel this environmental influence, the first frame from each capture stream has been subtracted, as shown in Figure 4e. However, even with start point normalization using a shift to the same position for all of the samples, the transient temperature responses are not monotonically related to the treatment period. To have the true rank of transient responses, our emissivity correction method has been adopted to generate Figure 4f. It presents the reconstructed transient thermal responses, which are based on adjusted emissivity. It shows that the induced thermal response decreases in amplitude as the case depth increases.

### 3.2. Fault Detection in Gears

To validate the proposed method for enhancing the detectability of the faults in real industrial applications, we have applied the method to a gear made of the same material in the case of carburized steels for crack visualization and failure detection. Pressure testing has been performed on this gear, and gear tooth 19 failed due to repeated contact pressing. A crack is generated across the gear tooth root and cannot be seen with the naked eye.

**Figure 5:**
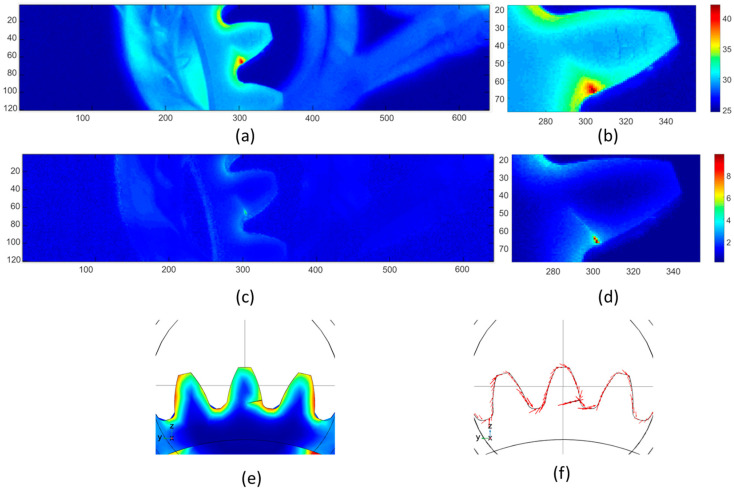
Thermal pattern reconstruction for fault detection in gears. (**a**) Raw data from thermography of failed gear tooth. (**b**) Enlarged image. (**c**) Emissivity corrected the thermal pattern of the gear. (**d**) Detailed failure distribution on the failed tooth. (**e**) Model design in COMSOL, Temperature responses at gear teeth, (**f**) Eddy current distribution.

Figure 5 shows the comparison of gear cracks of the original IR image and the reconstructed thermal pattern after emissivity correction at maximum heating frames. As seen in Figure 5a, the marked number 19 is visible in the thermal pattern due to different emissivity. A hot spot is located near the surface of the tooth root. As illustrated in Section 2, the hot spot is produced by the high density of eddy current induced at the crack. A reduced thermal gradient pattern is shown at the neighboring areas of the crack. Generally, this reduced thermal gradient pattern is due to heat transfer from the heat source (crack) to the surrounding materials. However, in this case, the thermal pattern cannot illustrate the true morphology of the crack due to various emissivity values across the surface. The crack can only be considered at the very small area of the hot spot near the surface. Based on the crack information provided by the testing service provider, a COMOSL model is built, as shown in Figure 5e, with the tested gear using a Helmholtz induction coil for investigating the eddy current distribution and thermal behaviors of the crack on gear without emissivity issues. Figure 5f shows the thermal responses of the gear and the high-temperature responses along the whole crack deep into the gear tooth. Figure 5f shows the eddy current distribution along the crack geometry at the fault position. The simulation results suggest that the crack deep into the materials should illustrate higher temperature responses than the surrounding materials. Therefore, the cracks shown in Figure 5a,b are highlighted by the concentrated induction heating, and the major thermal responses are the focus on the crack near the surface; it is hard to evaluate the propagation of the crack into the gear tooth. In this case, a reconstructed thermal pattern is obtained after applying the proposed emissivity correction algorithm. Figure 5c,d are the reconstructed thermal pattern after emissivity corrections. The shape of the crack is much clearer than it is in the original image. In Figure 5d, the heat responses are concentrated on the crack path, and the neighborhood areas are shown with uniformly low thermal behaviors. The distinguishing mark number 19 disappeared after the emissivity correction.

### 3.3. Gear Fatigue Analysis

**Figure 6:**
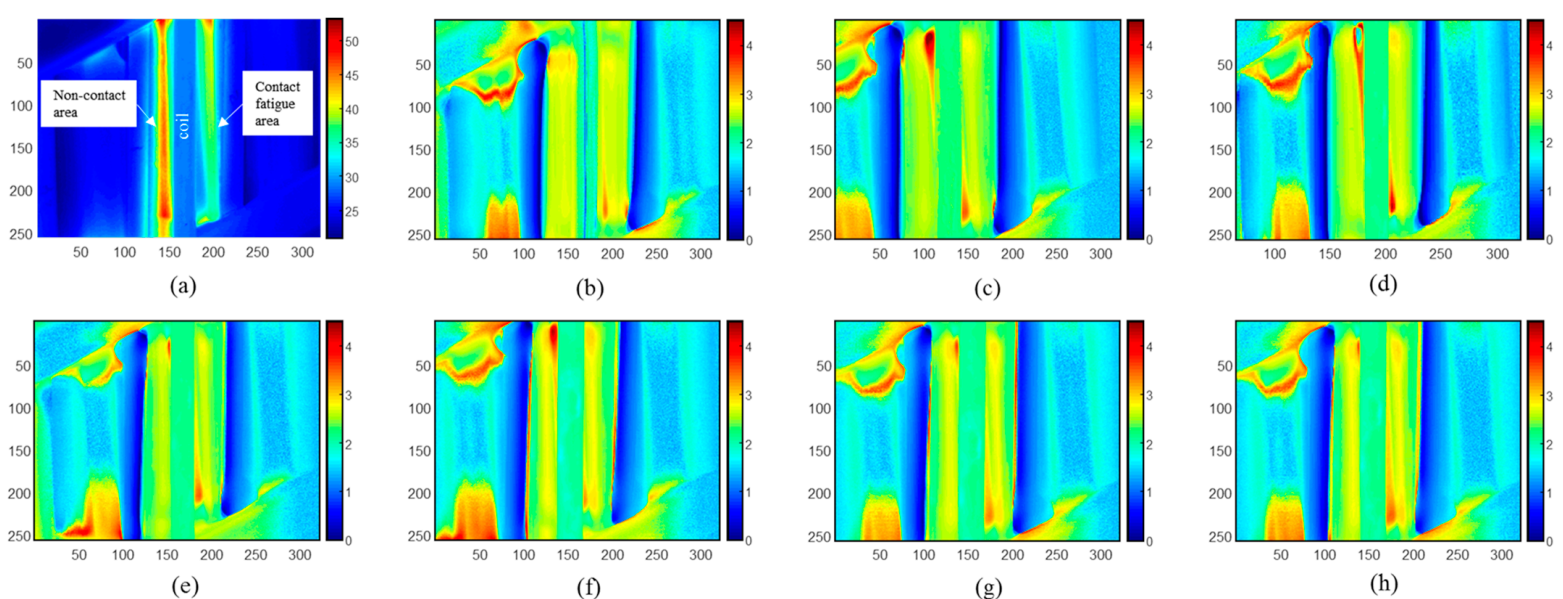
Fatigue evaluation after emissivity correction. (**a**) Raw IR image of stage 3. (**b**) Reconstructed image of stage 0. (**c**) Reconstructed image of stage 1. (**d**) Reconstructed image of stage 2. (**e**) Reconstructed image of stage 3. (**f**) Reconstructed image of stage 4. (**g**) Reconstructed image of stage 5. (**h**) Reconstructed image of stage 6.

As shown in Figure 6, an industrial gear was manufactured from 18CrNiMo7 heat-treated steels. The gears were tested on a 160 mm center distance back-to-back contact fatigue test rig at 3000 r/min (pinion) with BAG oil at 90 °C. A stepwise micro-pitting test involves running gears at incrementally increasing contact stress levels with each stage running for up to 8 × 106 cycles and having seven stages [15]. The 8 × 106 cycles take the running for 44.44 h, which takes approximately 160 × 103 s. Thermal videos were recorded for 2 s with a 400 ms heating stage and a 1600 ms cooling stage. The raw thermal pattern of the gear with 40 × 106 cycles (stage 3, start with stage 0) at 0.2 s is shown in Figure 6a. Fatigue and micro-pitting were developed on the contact surface during the fatigue test; fatigue development produces variations in thermal responses in spatial and transient domains [15]. During the transformation of retained austenite into martensite and fatigue softening, permeability increases as the number of cycles increases. After a certain number of cycles, dislocation accumulation will manifest which leads to a decrease in thermal conductivity and electrical conductivity [15]. Figure 6b–h illustrate emissivity-corrected results of gear from stage 0 to stage 6. Compared to the raw IR image, the emissivity issue in the brand-new gear at the initial state shown in Figure 6a is addressed in Figure 6b. Both sides of the tooth surfaces have the same thermal responses. Meanwhile, as seen in the contact fatigue area, the thermal pattern is divided into two major parts from Figure 6e–h. With the development of the fatigue process, the high thermal response areas at the contact fatigue area were separated, while the non-contact areas stayed the same.

## 4. Conclusions

In this paper, we proposed an efficient emissivity correction algorithm and thermal pattern reconstruction method based on the physical process modelling of active thermography. Based on the understanding of the mathematical and physical models, we proposed an emissivity correction method in eddy current pulsed thermography. By using this method, a reduction in the influence of non-uniform emissivity across the surface of a sample can be gained. For the first time, this paper demonstrated the analysis of materials and fault detections of heat-treated steels and gears using thermography with emissivity corrections. Experimental studies have been conducted in three aspects: (1) thermal pattern reconstruction for heat-treated steels based on emissivity correction and correction; (2) case-depth separation of the nitride steels with different times of heat treatment; (3) fault detection and fatigue process identification of heat-treated gears. Thermal pattern reconstruction can be achieved based on the modelling for enhancing the detectability of the faults in heat-treated gears with better contrast and detail textures; moreover, it can analyze the heat-treated materials in both manufacturing and fatigue scales. Although the emissivity-corrected thermal images significantly improved the accuracies of the thermal contract, there are still some highlighted pixels in area 1—as can be seen in Figure 3f, Figure 4c, and Figure 5d. This is due to the pixels having extremely high reflectivity and low emissivity, which leads to a low temperature response at the initial state. In this case, the numerator is much larger and the denominator is very small in Equation (11). This scenario provides uncertainties to the reconstructed thermal patterns in extreme conditions. The ability to analyze heat-treated materials in this technique potentially provide a huge benefit regarding improving detectability in high-speed-required NDT&E applications with different heat dissipation via geometry, such as in the rolling stock applications and other imaging and sensing scenarios [16,17,18,19,20].

## Data Availability

Data is unavailable due to privacy.
